# Effect of high vs. low volume of the nordic hamstring curl on hamstring muscle architecture and eccentric strength in soccer players: a systematic review and meta-analysis

**DOI:** 10.3389/fphys.2025.1631205

**Published:** 2025-10-16

**Authors:** Jozef Cholp, Erika Zemková

**Affiliations:** Department of Biological and Medical Sciences, Faculty of Physical Education and Sports, Comenius University in Bratislava, Bratislava, Slovakia

**Keywords:** injury prevention, football, knee flexors, muscle morphology, muscle force production

## Abstract

Hamstring strain injuries (HSI) remain a significant problem in professional soccer, as this injury is the most prevalent. Nordic hamstring exercise (NHE) is the most researched exercise regarding its effect on modifiable factors of HSI. However, there is still debate about the minimal effective dosage for this exercise. This systematic review and meta-analysis aimed (1) to analyse the effects of low- and high-volume NHE on eccentric strength and hamstring muscle architecture in soccer players, and (2) identify gaps in the literature to guide future research. Three electronic databases (PubMed, Web of Science, Scopus) were searched, and 11 studies met the inclusion criteria. Pooled effect sizes (Hedges’ g) and 95% confidence intervals (CI) were calculated using a random-effects model. High-volume NHE interventions significantly improved eccentric hamstring strength (g = 0.77, 95% CI 0.49–1.06, p < 0.001, I^2^ = 51%), fascicle length (g = 0.43, 95% CI 0.20–0.65, p < 0.001, I^2^ = 0%), and muscle thickness (g = 0.48, 95% CI 0.28–0.68, p < 0.001, I^2^ = 0%). Effects on pennation angle were non-significant (g = - 0.16, 95% CI -0.38–0.06, p = 0.16). Low-volume protocols significantly increased eccentric strength (g = 0.46, 95% CI 0.06–0.87, p < 0.05, I^2^ = 0%) but did not result in meaningful changes in fascicle length, pennation angle, or muscle thickness. For eccentric torque, neither high or low volume interventions produced significant effects (both g ≈ 0.04, p = 0.74, I^2^ = 0%). Control groups across all outcomes showed only trivial or negative changes. Results indicate that high volume of NHE (∼2–3 sets of 8–12 repetitions/2–3 times per week) significantly increases peak eccentric strength, fascicle length of biceps femoris long head, and muscle thickness, while pennation angle shows only trivial increase. Low volume of NHE (∼1–2 sets of 3–5 repetitions/1–2 times per week) shows a similar effect on peak eccentric hamstring strength, but there are no improvements in hamstring muscle architecture. Additionally, the effect of both types of volume on hamstring eccentric peak torque seems to be inconsistent. The variability of different testing methods on isokinetic strength and small correlations between other methods introduce challenges in comparisons with eccentric strength outcomes. A high volume of NHE seems to influence hamstring architecture adaptations better than low volume despite no differences in eccentric peak strength. Factors such as the player’s different level (amateur, semi-professional or professional), previous experience with NHE, and compliance significantly influence the training outcomes. Future research is needed to better determine the effect of low volume of NHE on the hamstring architecture adaptations in soccer players regarding previous experience with NHE and playing level. Furthermore, standardization of assessment tools and outcome measures is critical for future comparisons with isokinetic dynamometry.

## 1 Introduction

Muscle injuries are the most common in soccer at the elite (Ekstrand et al., 2011) and youth level (Palazon et al., 2022), accounting for nearly one-third of all time-loss injuries, with most of them affecting four major muscle groups of the lower limbs (Ekstrand et al., 2011). Hamstring strain injuries (HSI) have had the highest incidence (0.5 injuries/1,000 h of exposure) and injury burden over 30 years. They now make up roughly 19% of all reported soccer-related injuries, having increased from 12% to 24% over 21 consecutive seasons (2001–2002 to 2021–2022) (Ekstrand et al., 2023). These injuries have grown by 4% a year in elite soccer since 2001, with average time to return to play accounting for 17 days (Ekstrand et al., 2021). The biceps femoris long head muscle (BF^lh^) is involved in 84% of all first-time injuries, with semitendinosus (ST) injury resulting in ∼12% and semimembranosus (SM) about 4%. The etiology of HSI is multifactorial and complex in nature, and therefore it is inevitable to understand possible risk factors related to this type of injury. The previous HIS (Opar et al., 2012) and advanced age (Freckleton and Pizzari, 2013) have been considered as the main non-modifiable risk factors. Poor flexibility (Opar et al., 2012), low eccentric knee flexor strength, short muscle fascicles of BF^lh^ (Timmins et al., 2016), and core stability deficits (Schuermans et al., 2017) are considered as possible modifiable risk factors that contribute to a higher incidence of HIS. Most of the hamstring injuries (∼60%) take place during high-speed running actions (Woods et al., 2004) or sprinting (Ekstrand et al., 2021), while other mechanisms such as overstretch actions, shooting, or change of direction are also presented (Ekstrand et al., 2023). During high-speed running, the BF^lh^ is the muscle that lengthened the most despite possession of the shorter fascicles, opposite to BF^sh^ with longer fascicles (Kellis and Blazevich, 2022; Mao et al., 2024). This fact may explain the susceptibility of BF^lh^ to a higher risk of injury compared to other hamstring muscles (Thelen et al., 2005). The mechanism behind HSI seems to be the failure of the tissue to tolerate the forces applied while the task is performed (Cuthbert et al., 2020). The primary cause, however, has yet to be determined as the “weak link” approach, in which active lengthening (i.e., eccentric muscle action) of the sarcomeres creates a chronic accumulative cytoskeletal damage effect until the HSI occurs (Cuthbert et al., 2020).

The hamstring eccentric strength has been previously shown to play a crucial role in decreasing the risk of hamstring strain injury (Timmins et al., 2016). Decreasing eccentric strength accounts for 4.3%–5% higher injury risk (Opar et al., 2015). Absolute eccentric knee flexor strength with shorter fascicles in BF^lh^ significantly increased the risk of HSI in elite Australian soccer players (Timmins et al., 2016). Therefore, it appears that increasing muscle fascicle length along with eccentric strength of the hamstring muscles could potentially reduce the risk of HSI. ACWR (acute-to-chronic workload ratio) relative to lower or moderate ACWR is also associated with an increase in time-loss injury risk. A 2–4 times higher risk of injury for a player is presented when acute training load is 1.5 times higher than chronic workload (Gabbett, 2016).

Muscle architecture is considered to influence both force production and the velocity capabilities of the muscles (Lieber and Fridén, 2000). The muscle architecture mostly involves fascicle length (FL), angle of pennation (PA), muscle thickness (MT), or anatomical cross-sectional area (CSA). Muscle size (MT, CSA) can be influenced either by FL (i.e., length of fascicles between the aponeuroses/tendon) and PA (angle of fascicles relative to the tendon) or in reverse, which depends on the training mode. Traditional hypertrophy resistance training is responsible mainly for the increasing of muscle CSA with an increase in PA (Aagaard et al., 2001) and a modest or no increase in FL (Seynnes et al., 2007; Franchi et al., 2014). On the other hand, eccentric training appears to have a greater effect on the increase of muscle CSA (Douglas et al., 2017) and FL (Franchi et al., 2014; Gérard et al., 2020) opposite to hypertrophy resistance training. Some evidence assumes that eccentric training can increase muscle fascicle length of the BF^lh^ muscle (Potier et al., 2009) and increase muscle PA of the vastus lateralis muscle (Guilhem et al., 2013).

The Nordic hamstring exercise was first documented when evaluating its acute effect on the angle of peak torque of the hamstrings during eccentric isokinetic testing (Brockett et al., 2001). Since then, the NHE has been demonstrated to be an effective injury prevention method, as it enhances (Mjølsnes et al., 2004; Arnason et al., 2008; Opar et al., 2012) and evaluates hamstring eccentric strength (Opar et al., 2013) alongside the increase of BF^lh^ FL (Timmins et al., 2016). This exercise provides a slow eccentric stimulus where myosin heads are already attached to actin, where, due to the eccentric nature of the movement, they are forced to detach by lengthening of the cross-bridges, which leads to significant damage in the muscles (Franchi et al., 2017). This exercise provides eccentric overload, where the hamstrings must perform their maximal eccentric force production. High muscle damage to the muscles is incurred, which subsequently may result in delayed onset muscle soreness (DOMS) of the muscles involved (Lieber and Jan, 2002). Some authors assume that the NHE program implementation at the highest levels of professional soccer is too low and therefore is not expected to have an overall effect on acute hamstring injury rates because of the high occurrence of DOMS in players (Bahr et al., 2015). Although the NHE has been shown to be an effective strategy for injury prevention of HSI incidence in many sports, practitioners still disagree about whether a low or high volume of the NHE is best suited for improving the modifiable risk factors of HSI (Medeiros et al., 2021).

No significant difference was shown in a systematic review for the effects of applying a high volume of this exercise versus a low volume on the eccentric hamstring muscle strength or the length adaptations of the biceps femoris fascicle (Cuthbert et al., 2020). On the other hand, this meta-analysis includes articles with a mixed population (professional athletes, amateur athletes, and recreationally active people), which results in high variability from a strength level and muscle architecture perspective (Cuthbert et al., 2020). Focusing on one specific group where the NHE application is the most prevalent may give the practitioners more applicable information about the usage of this exercise. The aim of this systematic review and meta-analysis are to (1) investigate how the architecture (PA, FL, MT) and eccentric strength of the knee flexor muscles are affected by either high or low volumes of NHE in amateur, semiprofessional, and professional soccer players; (2) identify gaps in the current literature and propose future research on this topic.

## 2 Methods

### 2.1 Study design

This systematic review was designed in accordance with the Preferred Reporting Items for Systematic Reviews and Meta-Analyses (PRISMA) guidelines. The PRISMA statement provides a 27-item checklist intended to guide the reporting of systematic reviews, particularly those involving randomized controlled trials (Page et al., 2021).

### 2.2 Literature search

To search all relevant studies, three electronic databases were chosen (PubMed, Web of Science, and Scopus). For additional search, the backward search was used (i.e., assessing the reference lists of included articles). The terms such as “Nordic curl” and “Nordic hamstring” were combined with the terms “football” and “volume” to find any title and abstract that is related to our topic. Only publications in the English language were included. Boolean terms “AND” and “OR” were used for the keyword’s combination.

### 2.3 Inclusion and exclusion criteria

The main inclusion criteria were that studies had to examine the NHE effect of either high or low volume or a comparison of both on muscle architecture adaptations and/or eccentric strength variables of hamstring muscles in amateur, semi-professional, or professional soccer players of both genders. Other criteria were based on the publication date of 2014–2025 and full text availability of the articles. The duration of the included study had to be more than 4 weeks, and the experimental group could not have possessed any lower limb injury at least 6 months before the start of the study. The muscle architecture had to be measured on BF. Mean ± standard deviation (SD) pre- and post-intervention were provided for the measured variables for secondary analysis. Additionally, there had to be an absence of any health disorders that would interfere with the study results. The next criteria were based on assessing the post-PHV players (peak height velocity) to prevent the influence of the maturation status on our results (Drury et al., 2020). Studies were excluded if they collected data solely through injury incidence questionnaires without measuring physiological or performance adaptations. Additionally, isokinetic data collected at angular velocities greater than 120°/s were excluded due to decreased reliability and a reduced percentage of the range of motion maintained at a constant velocity as angular speed increases (Kellis and Baltzopoulos, 1995).

If the studies failed to meet our criteria, they were excluded from this review. It should be mentioned that although women may possess lower peak strength values than men (Medeiros et al., 2021), we decided to include the participants of both genders, as our analyses considered the strength and architecture adaptation differences between pre- and post-intervention rather than peak strength values of athletes (Medeiros et al., 2021).

### 2.4 Quality and risk of bias assessment

The methodological quality of the studies was assessed using the Testex scale (Smart et al., 2015), a quality assessment instrument specifically designed for exercise training studies. It assesses methodological quality based on 12 criteria, with a total possible score of 15 points. Reference scores were used to express the quality level of the studies and are presented as follows: <4 points “poor quality,” 4–7 points “moderate quality,” 8–10 points “good quality,” and >11 points “excellent quality” (Davies et al., 2021). Two types of bias assessments were carried out in this review: the Rosenthal fail-safe N method (Rosenthal, 1979) and the Cochrane risk of bias tool for randomized controlled trials (Sterne et al., 2019). The Cochrane tool evaluates RCT’s across several domains, such as the randomization process, deviations from the intended intervention, missing outcome data, etc. Each domain is rated as having a “low risk of bias,” “high risk of bias,” or “some concerns.” The fail-safe N was applied to estimate how many unpublished or missing studies with null results would be required to reduce the observed effect to nonsignificance (p > 0.05).

### 2.5 Analysis and interpretation of results

Means and standard deviations for strength outcomes and muscle architecture measures were independently extracted from the included studies. Strength outcomes included isokinetic assessments such as eccentric peak torque (measured at 30°/s, 60°/s, and 120°/s) and eccentric force. Muscle architecture variables included fascicle length, pennation angle, and muscle thickness.

Effect sizes (ES) were calculated to provide standardized comparisons of mean differences between groups or experimental conditions. Hedges’ g, along with 95% confidence intervals (CI), was used to quantify mean differences between pre- and post-intervention, as it adjusts for sample size disparities. Hedges’ g was determined using the following formula (Hedges and Ingram, 1985):

$$g=\frac{\left(Mean\;post-Mean\;pre\right)}{\text{SD}\;\text{pooled}}$$

Interpretation of ES was followed by Hopkins thresholds (Hopkins, 2010): trivial (≤0.20), small (0.20–0.59), moderate (0.60–1.19), large (1.20–1.99), and very large (≥2.00). Consistency across studies was assessed using Higgins test for heterogeneity (I^2^) (Hopkins, 2010), with values interpreted as low (<25%), moderate (25%–75%), or high (≥75%) heterogeneity. Between-study variance estimates for both strength and architecture outcomes were calculated using random-effects models, with results expressed as 95% confidence intervals (The Jamovi project, 2019). Effect sizes and random-effects models were calculated using Jamovi software (version 2.7.6). For clarity and improved visualization, forest plots were created separately based on the extracted effect size data.

## 3 Results

### 3.1 Search results

**FIGURE 1:**
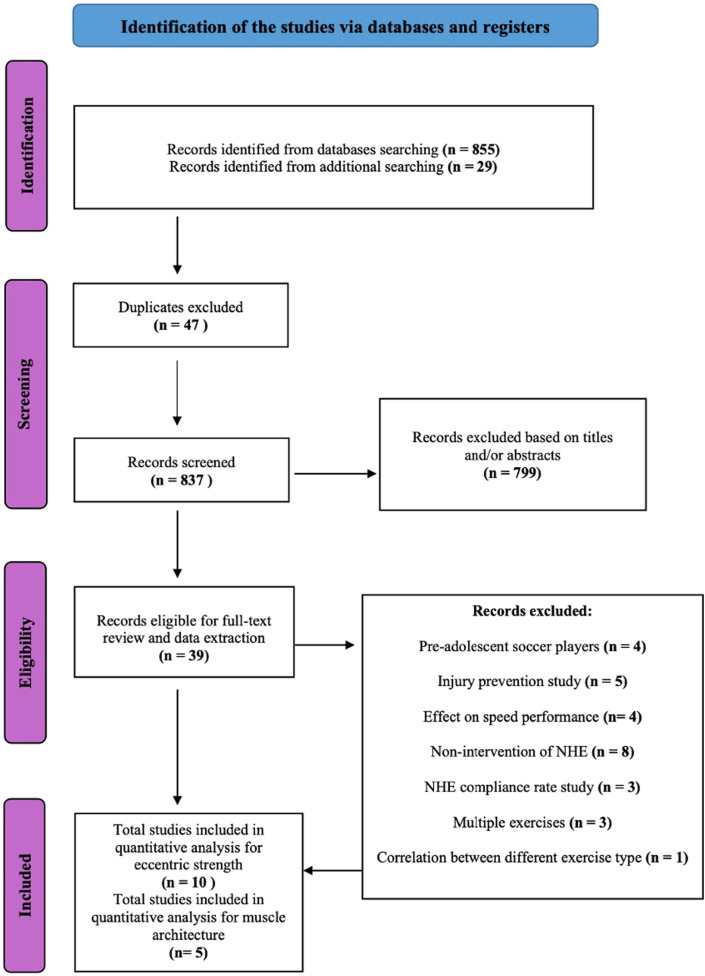
Search of NHE intervention studies.

The PRISMA flow diagram (Figure 1) illustrates the search and selection stages (Page et al., 2021). Initially, we identified 855 articles from the main databases and another 29 articles, which provided an additional backward search from the reference lists of relevant studies. After the exclusion of duplicates (n = 47), we removed studies that did not research hamstring strength variables or architectural adaptations after NHE intervention in a soccer population. Thirty-nine relevant articles were included in the full-text analyses, and eleven of them reported suitable data for quantitative analysis. The outcomes extracted from the studies were eccentric knee flexor strength or eccentric knee flexor torque. Other outcomes obtained from the studies were fascicle length (FL), pennation angle (PA), and muscle thickness (MT) of BF^lh^, SM, and ST muscles, which represent the hamstring muscle architecture. For qualitative analysis Pre-post differences were calculated from mean values and expressed as percentages. These indicators serve to improve the understanding of the collected data (Table 3). For quantitative analysis, data were collected from the interventions reported in the included studies, comparing outcomes before and after the interventions. Information from both interventions or/and control groups was extracted when available; however, having a control group was not part of the inclusion or exclusion criteria. As a result, three of the included studies, de Oliviera et al. (2020), Vianna et al. (2021), and Siddle et al. (2024) did not include control groups.

### 3.2 Study quality and bias results

Quality of assessment was assessed using TESTEX criterion. The mean score of the included studies was 8 out of 15 points, with the highest-scoring studies being randomized trials (Lovell et al., 2018; Medeiros et al., 2020). The scores in 6 of the presented studies were of moderate quality, 3 studies were of good quality, and 2 studies were presented with excellent quality scores (Table 1).

**TABLE 1:** Study scores allocated based on TESTEX criteria.

Study	1 (1 point)	2 (1 point)	3 (1 point)	4 (1 point)	5 (1 point)	6 (3 points)	7 (1 point)	8 (2 points)	9 (1 point)	10 (1 point)	11 (1 point)	12 (1 point)	Total
de Oliviera et al. (2020)	1	0	0	1	0	1	0	1	1	0	0	1	6
Suarez-Aronnes et al. (2019)	1	1	0	1	0	1	0	2	1	0	0	1	8
Cadu et al. (2022)	1	0	0	1	0	1	0	2	1	0	0	1	7
Amundsen et al. (2022)	1	1	0	1	0	1	0	2	1	0	1	1	9
Medeiros et al. (2020)	1	1	0	1	1	3	1	2	1	0	1	1	13
Lovell et al. (2018)	1	1	0	1	1	3	1	1	1	1	1	1	13
Ishoi et al. (2018)	1	1	1	1	1	1	0	2	1	0	1	1	11
Sebelien et al. (2014)	1	1	0	1	0	1	0	0	1	0	0	1	6
Vianna et al. (2021)	1	0	0	1	0	1	0	1	1	0	0	1	6
Siddle et al. (2024)	1	0	0	1	0	1	0	1	1	0	0	1	6
Mendiguchia et al. (2020)	1	0	0	1	0	1	0	2	1	0	0	1	7

1. Eligibility criteria specified; 2. Randomization specified; 3. Allocation concealment; 4. Groups similar at baseline; 5. Blinding of assessor 6. Outcome measures assessed in 85% of subjects; 7. Intention-to-treat analysis; 8. Between-group statistical comparisons reported; 9. Point measures and measures of variability of outcomes reported; 10 Activity monitoring in control groups reported relative exercise intensity retained constant; 12. Exercise volume and energy expenditure.

For the risk of bias, two tools were also performed. The Cochrane risk of bias (Table 2) shows an overall “some concerns” for the review, mostly due to unclear randomization methods or incomplete reporting of missing data. The second tool identifies that the results of this meta-analysis are not subject to publication bias (p < 0.001), with 178 “filed away” studies for eccentric strength and 30 studies for muscle architecture needed to prove null effects of NHE interventions on eccentric strength and muscle architecture. These results indicate that the findings for eccentric strength are robust and unlikely to be overturned by unpublished null studies. In contrast, the muscle architecture outcomes appear more fragile, as relatively few unpublished studies could change the statistical significance.

**TABLE 2:** Cochrane risk of bias for randomization controlled trials.

Study	Randomization process	Deviations from intended intervention	Missing outcome data	Measurment of outcome	Selection of reported result	Overall RoB
Ishoi et al. (2017)	Low risk 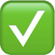	Low risk 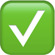	Low risk 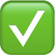	Low risk 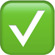	Low risk 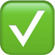	Low risk 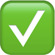
Suarez-Aronnes et al. (2019)	High risk 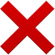	Low risk 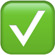	Low risk 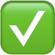	Low risk 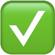	Some concerns 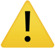	High risk 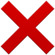
Mendiguchia et al. (2020)	Some concerns 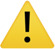	Low risk 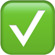	Low risk 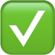	Low risk 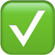	Some concerns 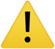	Some concerns 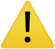
Sebelien et al. (2014)	Some concerns 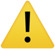	Low risk 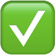	Low risk 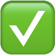	Low risk 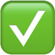	Some concerns 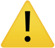	Some concerns 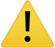

### 3.3 Systematic review and meta-analysis findings

**FIGURE 2:**
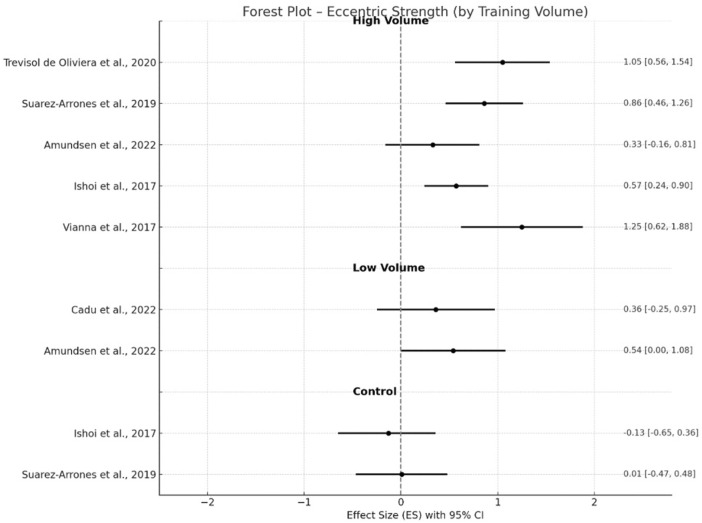
Changes in eccentric strength pre- and post- NHE intervention.

**FIGURE 3:**
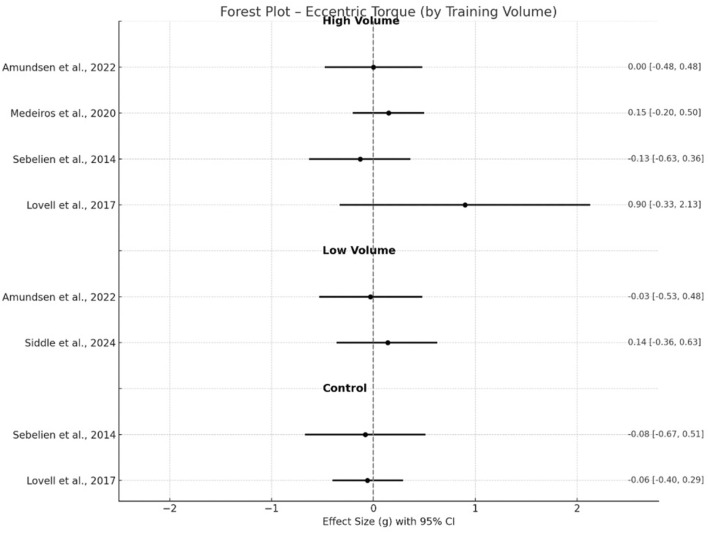
Changes in eccentric torque pre- and post- NHE intervention.

**FIGURE 4:**
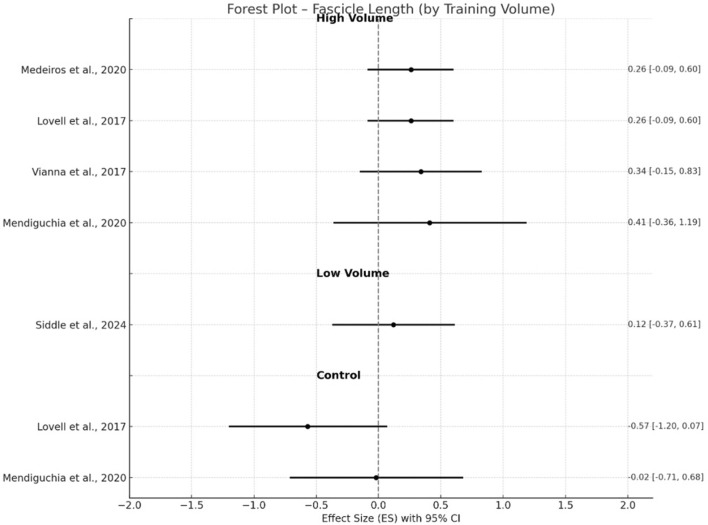
Changes in fascicle length pre- and post- NHE intervention.

**FIGURE 5:**
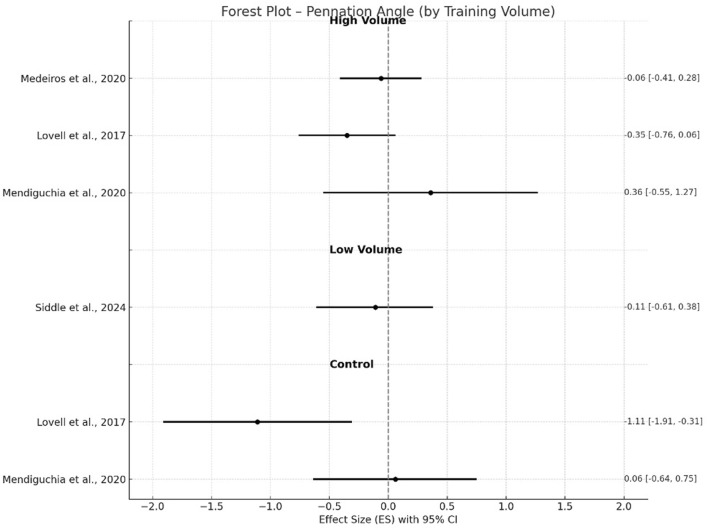
Changes in pennation angle pre- and post- NHE intervention.

**FIGURE 6:**
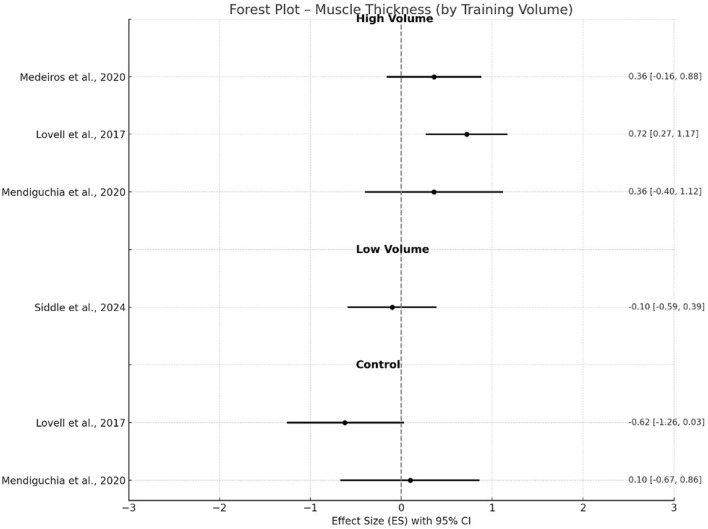
Changes in muscle thickness pre- and post- NHE intervention.

Within-study pre–post differences showing the magnitude of change (Hedge’s g, 95% CI) across all included trials are illustrated in Figures 2–6 below. For eccentric hamstring strength, high-volume interventions (de Oliviera et al., 2020; Suarez-Arrones et al., 2019; Amundsen et al., 2022; Ishøi et al., 2018; Vianna et al., 2021) consistently demonstrated moderate improvements (g = 0.77, p < 0.001, 95% CI 0.49–1.06), with moderate heterogeneity (I^2^ = 51%). Low-volume protocols (Cadu et al., 2022; Amundsen et al., 2022) also produced significant but small effects (g = 0.46, p < 0.05, 95% CI 0.06–0.87) with no heterogeneity (I^2^ = 0%), while control groups (Suarez-Arrones et al., 2019; Ishøi et al., 2018) showed only trivial or negative effects (g = −0.06, p = 0.75, 95% CI −0.40–0.29), with moderate heterogeneity (I^2^ = 51%). In the case of eccentric torque, low volume interventions (Amundsen et al., 2022; Siddle et al., 2024) did not result in significant gains, with only trivial effect (g = 0.04, p = 0.74, 95% CI −0.21–0.29), as well as high-volume interventions (g = 0.05, p = 0.55, 95% CI −0.12–0.23) (Medeiros et al., 2020; Amundsen et al., 2022; Sebelien et al., 2014; Lovell et al., 2018). No heterogeneity (I^2^ = 0%) was presented in low and high volume groups, respectively. Two studies provided a control group (Sebelien et al., 2014; Lovell et al., 2018), resulting in a trivial negative effect (g = −0.07, p = 0.67, 95% CI −0.67–0.51, I^2^ = 0%).

Changes in muscle architecture were more variable across outcomes. For fascicle length, high-volume interventions (Medeiros et al., 2020; Lovell et al., 2018; Vianna et al., 2021; Mendiguchia et al., 2020) elicited significant changes with a small effect size (g = 0.43, p < 0.001, 95% CI 0.20–0.65) and no heterogeneity (I^2^ = 0%), whereas control groups (Lovell et al., 2018; Mendiguchia et al., 2020) showed a small negative effect (g = −0.31, p = 0.25) with low heterogeneity (I^2^ = 23.7%). Low volume intervention (Siddle et al., 2024) resulted in non-significant gains with only trivial ES (g = 0.12, 95% CI −0.37–0.61).

In contrast, adaptations in pennation angle were not statistically significant (g = −0.16, p = 0.16, 95% CI −0.38–0.06, I^2^ = 0%) with trivial to small negative ES for either high-volume groups (Medeiros et al., 2020; Lovell et al., 2018; Mendiguchia et al., 2020) or control groups (g = −0.51, p = 0.39, I^2^ = 78.6%) (Lovell et al., 2018; Mendiguchia et al., 2020). Low volume group (Siddle et al., 2024) again resulted in no significant changes (g = −0.11, −0.61–0.38) after the training intervention. For muscle thickness, high-volume interventions (Medeiros et al., 2020; Lovell et al., 2018; Mendiguchia et al., 2020) demonstrated significant increases with a small effect (g = 0.48, p < 0.001, 95% CI 0.28–0.68), whereas control groups showed a moderate negative effect (g = −0.91, p = 0.39) with high between-study heterogeneity (I^2^ = 88.3%). A trivial negative effect (g = −0.10; 95% CI −0.59–0.39) was found in low volume group (Siddle et al., 2024).

## 4 Discussion

### 4.1 Eccentric strength adaptations to NHE of varying volumes

Although the NHE has been widely used by strength and conditioning coaches, no consensus about dosage and its manipulation in training has been reached yet. There was a wide range of volumes and durations of included studies from our literature search (Table 3). In summary, 10 of 11 articles included the measuring of eccentric strength variables of hamstring muscles. The high volume of NHE has been performed in 8 studies (de Oliviera et al., 2020; Amundsen et al., 2022; Suarez-Arrones et al., 2019; Ishøi et al., 2018; Vianna et al., 2021; Lovell et al., 2018; Sebelien et al., 2014; Medeiros et al., 2020), whereas only 3 articles applied a low volume of NHE (Amundsen et al., 2022; Cadu et al., 2022; Siddle et al., 2024). Closer insights into the training program and results of included studies are presented in Table 3.

**TABLE 3:** Characteristics of the studies with NHE intervention in soccer players.

Authors	Study design	Participants Age (years) Number (n) NHE experience	Duration (weeks)	Procedures	Compliance rate (%)	Training intervention	Results
de Oliviera et al. (2020)	Quasi experimental study	Male soccer players (18.32 ± 0.63 years) n = 25 Not trained in NHE	4 weeks	Peak eccentric hamstring strength (N)	100%	2x per week 3 x 6–10 reps Total volume = 192 reps (High volume)	-Significant improvements in peak eccentric strength -∆ Pre - Post (+13 ± 9.6 %), p < 0.001 -ES (Cohen‘s d) = 0.95
Suarez-Arrones et al. (2019)	Randomized controlled trial	Male professional soccer players n = 50 (18.8 ± 0.8 years) Not trained in NHE (NG1) Trained in NHE (NG2)	Nordic group 1 (NG1) = 17 weeks Nordic group 2 (NG2) = 15 weeks	Peak absolute eccentric hamstring strength (N) Peak relative eccentric hamstring strength (N/kg)	(n.d)	Week 1–15/17 = 1–2 x per week/2–3 x 6–10 reps Total volume NG1 = 538 reps NG2 = 598 reps (High volume)	-Significant impovements in NG1 (Nordic group 1) -∆ Pre - Post (+16.5 ± 5.6 %), p < 0.01 -ES = 0.80 ± 0.26 -Significant improvements in relative peak eccentric strength in NG1 -∆ Pre - Post (+14.7 ± 5.7 %), p < 0.01 -ES; ± 90 CL = 0.74 ± 0.26 -No improvements in NG2 for any measurment
Cadu et. al. (2022)	Randomized trial	Profesional male soccer players (25.6 ± 3.5 years) n = 23 Not trained in NHE	21 weeks	Peak eccentric hamstring force (N)	47.2 % Both groups (High, low compliance)	Week 1–21 = 1x per week/1x3 reps Additional weight if possible Total volume = 63 reps (Low volume)	-Significant increase after the training program in both experimental groups - ∆ Pre - Post (+15.5 ± 25.5 %), p = 0.049 -ES (Hedge g) = 0.42 -Significant improvements in peak eccentric strength for “high compliance”group (+15.5 %), CI [95 %] (1.2 – 29.8); p < 0.001 -ES (Hedge g) = 1.2
Amundsen et al. (2022)	Randomized trial	Female professional soccer players Low volume group (20 ± 2 years) n= 15 High volume group (21 ± 4 years) n = 17 Not trained in NHE	8 weeks	Peak eccentric hamstring strength (N) Eccentric knee flexion peak torque 60°/s (N.m)	High volume group: 89% Low volume group: 93%	High volume group Week 1–8 = 1–3x per week/2–3 x 5–12 reps Low volume group Week 1–8 = 1–2x per week/2–4 x 4–6 reps Total volume HV = 538 reps LV = 144 reps	-Significant increase in eccentric strength for high volume group -∆ Pre - Post (+10 %), p < 0.001, CI [95%] (19 – 38 N) -Significant increase in eccentric strength for low volume group -∆ Pre - Post (+13 %), p = 0.001, CI [95%] (18 – 55 N) -No between group differences in eccentric hamstring strength, p = 0.38 - No significant increase in eccentric peak torque for high volume group, p = 0.88; low volume group p = 0.54
Medeiros et al. (2020)	Randomized trial	Professional male soccer players n (G1) = 15 (18.8 ± 1.74 years) n (G2) = 17 (18.5 ± 1.1 years) Not trained in NHE	8 weeks	Eccentric peak torque (N.m^−1^) (60°/s) BF^lh^ MT (cm) BF^lh^ PA (°) BF^lh^ FL (cm)	100%	G1 = 1 x per week/2–4 x 6–10 reps G2 = 2 x per week/2–4 x 6–10 reps Total volume G1 = cca. 250 reps (High volume) G2 = cca. 456 reps (High volume)	- No significant increase in eccentric peak torque for G1 - Significant increase in eccentric peak torque for G2 -∆ Pre - Post (+19 N.m^−1^), CI [95 %] = 212–234; ES = 0.52 -MT, FL increased in group 1x week (moderate, small ES); no increase PA -MT, FL increased in group 2x week (small ES); no increase PA -No significant difference between groups
Lovell et al. (2018)	Randomized trial	Amateur male soccer players n = 31 (23.6 ± 4.7 years) Not trained in NHE	12 weeks	Eccentric knee flexor peak torque (N.m^−1^) (30°/s) BF^lh^MT (cm) BF^lh^PA (°) BF^lh^FL (cm)	NHE _BEF_= 34.7% NHE_AFT_= 46.8%	Week 1–12 = 1–2 x per week/2–4 x 5–12 reps Total volume for NHE BFT and NHEAFT group = 684 reps (High volume)	-Significant changes in peak eccentric torque for both groups -NHE_BEF_= -∆ Pre - Post (+11.9%), CI [90%] (3.6–20.9%) NHE_AFT_= −∆ Pre - Post (+11.6 %), CI [90%] (2.6 – 21.5%) -MT increased in the NHE_AFT_–∆ Pre - Post (+0.17 cm), CI [90%]: (0.05–0.29 cm), Medium ES - PA increased in the NHE_AFT_ -∆ Pre - Post (+1.03°), CI [90 %] (−0.08–2.14°), small ES - FL increased in the NHE_BEF_–∆ Pre - Post (+1.58 cm), CI [90%] (0.48–2.68°), small ES
Ishoi et al. (2018)	Randomized controlled trial	Amateur male football players n = 10 (19.1 ± 1.8 years) Not trained in NHE	10 weeks	Peak eccentric hamstring strength (N)	60.2%	Week 1–10 = 1–3x per week/2–3x 6–12 reps Total volume for IG = cca. 700 reps (High volume)	-Significant improvements in peak eccentric strength for experimental group -∆ EG - CG (+62.3 N), p < 0.01, CI [95 %] = (20.0 – 104.5 N) -ES (Cohen’s d) = 0.92
Sebelien et al. (2014)	Randomized controlled trial	Semi-professional male soccer players n = 16 (20–36 years) Not trained in NHE	10 months	Eccentric knee flexor peak torque (N.m^−1^) (60°s^−1^)	27.7%	Week 1–5 = Not specified/2–3x 5–12 reps Total volume after 5 weeks = cca. 232 reps (High volume)	-No significant changes in eccentric peak torque for experimental group
Vianna et al. (2021)	Quasi experimental trial	Professional female soccer players n = 17 (24 ± 5 years) Not trained in NHE	8 weeks	Peak eccentric hamstring strength (N) BF^lh^FL (cm)	100%	Week 1–8 = 2x per week/2–4x 6–10 reps Total volume = cca. 456 reps (High volume)	- Significant increase in peak eccentric strength -∆ Pre - Post (+13.6%), CI [95 %] (268 – 320), p < 0.01; ES = 0.78 -Significant increase in BF^lh^fascicle length -∆ Pre - Post (+ 6.4%), CI [95%] (9.8–11), p < 0.001; ES = 0.56
Siddle et al. (2024)	Quasi experimental study	Elite male academy soccer players n = 16 (16.7 ± 0.6) Previously trained in NHE	8 weeks	Eccentric knee flexor peak torque (N.m^−1^) (60°/s, 180°/s, 270°/s) BF^lh^ MT (cm) BF^lh^ PA (°) BF^lh^ FL (cm) SM MT (cm) ST MT (cm)	93%	Week 1–2 = 2x per week/4x6 reps Week 3–8 = 1x per week/2x4 Total volume = 144 reps (Low volume)	-No significant increase in peak torque in any of test speed, p = 0.19 - No significant difference in MT BF^lh^, p = 0.44 - No significant difference in MT SM, p = 0.30 - No significant difference in MT ST, p = 0.48 -No significant difference in PA BF^lh^, p = 0.56 -No significant difference in FL BF^lh^, p = 0.57
Mendiguchia et al. (2020)	Randomized controlled trial	Professional male soccer players n = 7 (n.d) Not trained in NHE	6 weeks	BF^lh^ MT (cm) BF^lh^ PA (°) BF^lh^ FL (cm)	100%	Week 1–6 = 1–3x per week/2–3x 5–12 reps Total volume = cca. 340 reps (High volume)	-Likely small increase in BF^lh^ FL after intervention -∆ Pre - Post (+7.38 ± 4.03 %) ES; ± 90 % CL = 0.58 ± 0.33 - Possibly small increase in BF^lh^ PA after intervention -∆ Pre - Post (+ 9.24 ± 8.60 %) CL= 0.41 ± 0.62 - Likely small increase in BF^lh^ MT after NHE intervention -∆ Pre - Post (+ 5.04 ± 2.11) ES; ± 90 CL = 0.46 ± 0.40

BF^lh^ (Biceps femoris long head); ST (Semitendinosus); SM (Semimembranosus); MT (Muscle thickness); PA (Pennation angle); FL (Fascicle length); NHEBEF (Before session); NHEAFT (After session); NHE (Nordic hamstring exercise); HV (High volume); LV (Low volume); ± 90 CL (Confidence limit); CI [90–95%] (Confidence interval); ES (Effect size); n.d. (No data).

Performing high volumes of NHE leads to a significant increase in peak eccentric strength after 4 weeks (de Oliviera et al., 2020) and after longer periods (>8 weeks) (+10%–16.5%). (Amundsen et al., 2022; Vianna et al., 2021; Suarez-Arrones et al., 2019; Ishøi et al., 2018; Medeiros et al., 2021). All studies, where peak eccentric strength was investigated, found significant gains after high volumes of NHE. However, in a study where the players had been previously trained using NHE, no significant increase was seen (Suarez-Arrones et al., 2019). In addition, a high volume of NHE leads to significant improvements in eccentric peak torque only in 2 (Lovell et al., 2018; Medeiros et al., 2020) of 4 studies (Lovell et al., 2018; Medeiros et al., 2020; Amundsen et al., 2022; Sebelien et al., 2014).

Low volume of this exercise leads to a significant increase of peak eccentric strength in female soccer players (Amundsen et al., 2022), with no significant differences between low and high volume groups. Significantly higher peak eccentric strength was found after performing a very low volume (1 set of 3 reps) for 21 weeks and with a low compliance rate (47.2%) and with higher changes for the “high compliance subgroup” (Cadu et al., 2022). Two studies in our review examined the effect of low volume on eccentric peak torque in professional female (Amundsen et al., 2022) and academy soccer players (Siddle et al., 2024) with no significant gains.

The variability of results in increasing eccentric torque after NHE can be explained by the poor correlation between peak eccentric strength measured on NordBord and eccentric peak torque measured on isokinetic dynamometers (Amundsen et al., 2022; Wiesinger et al., 2020). This fact can be explained by the low similarity of movement when performing tests on the NordBord device versus the isokinetic machine (Medeiros et al., 2021). Furthermore, any significant correlations between the eccentric peak torque during NHE and peak eccentric knee flexion torque (r = 0.24–0.3, p = 0.26–0.4) were found when measured on a NordBord and isokinetic dynamometer (Nishida et al., 2022). Similarly poor correlation (r = 0.35) was found between eccentric force during NHE and isokinetic eccentric peak torque at 60°/s (Van Dyk et al., 2018). Another issue seems to be the difference in the body positions (prone, sitting) and different movement velocities (30°/s, 60°/s, 180°/s, 270°/s) that were used in the studies to measure this parameter. Significant changes were observed after performing a high volume of NHE in 2 studies (Lovell et al., 2018; Medeiros et al., 2020), but only when using lower angular velocity during testing on isokinetic devices (30°/s, 60°/s). Measuring eccentric peak torque has therefore seemed to be highly influenced by other factors such as the movement performed and the velocity, which must be taken into consideration. On the contrary, emphasizing higher angular velocities during testing with an isokinetic dynamometer seems to have some applicability, as sprinting is the most frequent action during which HSI is occurring. The use of high velocities can better explain specific hamstring work during this task, although it is performed in isolation.

The peak eccentric strength measured during NHE on the NordBord device (Opar et al., 2013) is the most used variable in practice and research. However, there are some misconceptions about which variable is the best for assessing the eccentric hamstring strength. Opar et al. (2015) suggest implementing torque measurement during NHE on NordBord, as there are some differences between athletes with longer or shorter lower leg levers. Despite this, they claim that measuring the force output during NHE still provides useful information for HSI risk. Buchheit et al. (2016), on the other hand, found that measuring the knee flexor strength is largely body mass dependent, but simply dividing absolute strength by athlete body mass is not a sufficient method. They assume to use the provided equation (eccentric strength [N] = 4 x BM [kg] + 26.1) for the estimation of players’ expected strength based on their own body mass (BM) and compare it to their actual peak force value from the test when monitoring players over longer periods when BM changes may occur. This equation was developed to differentiate what effect the athlete’s BM had compared to his true eccentric strength. On the other hand, Opar et al. (2021) did not find any difference between prospectively injured or non-injured soccer players irrespective of the quantification tool used (between-limb asymmetry, relative or absolute strength) in pre-season eccentric strength. Therefore, practitioners can use different quantifications of eccentric strength, based on their goal.

Only 6 of 11 investigated studies provided a compliance rate greater than 80%. Some previous studies (Chesterton et al., 2021) have shown that the low compliance rate in soccer players is a result of DOMS presenting after the NHE. Goode et al. (2015), in their meta-analysis, found that the effect of the exercise is also highly influenced by intervention compliance. Similar results were reported by Rudisill et al. (2023) and Biz et al. (2021), who demonstrated that eccentric hamstring training, particularly the NHE and other injury prevention protocols, can reduce hamstring injury incidence by up to 70%, but poor compliance and limited implementation in team practice remain major barriers to effectiveness.

The groups where a high volume of NHE has been performed increased intensity via increased volume, whereas in low volume groups it is more likely that the increase in intensity was due to increasing the breakpoint angle of the hamstrings during movement because no volume has been increased (Cuthbert et al., 2020). The athlete can last longer and get his torso closer to the ground, which increases the torque due to force being applied over a greater momentum. For improving muscle strength, the performed intensity must be over 85% of 1 repetition maximum (RM) and ∼6 reps (Haff et al., 2016). This mostly applies to more advanced athletes, whereas in novice athletes, lower intensity is also preferred to elicit improvements in strength capabilities. The NHE is supramaximal in nature, and intensity is above 1RM; therefore, it applies true eccentric stimulus on the hamstring muscles. The assumption would be that lower repetitions are enough to stimulate hamstrings at the same level. Severo-Silveira et al. (2021), on the other hand, found that a progressive workload (236 reps over 8 weeks) had better results than a constant workload (138 reps over 8 weeks) from an eccentric strength perspective.

From the results of our studies, it seems that a lower volume of this exercise can be as sufficient as high volume in improving the peak eccentric hamstring strength measured on a NordBord. On the contrary, the effect of both volume training types on eccentric peak torque seems to be inconsistent in findings. The variability of different testing methods on isokinetic dynamometry and small correlations with other methods introduce challenges in comparing eccentric strength outcomes. Practitioners must consider the low validity of NHE when measuring the eccentric strength on an isokinetic device.

### 4.2 Hamstring architecture adaptations to NHE of varying volumes

In recent decades, the introduction of 2D image ultrasound has allowed the study of muscle architecture (Blazevich et al., 2006). This cost-effective and time-saving noninvasive method has helped to expand the assessment of muscle thickness, pennation angle, and fascicle length, particularly in the BF^lh^, which is the most researched muscle regarding the HSI. However, the use of two-dimensional ultrasound in estimating fascicle length has been presented with some methodological limitations. Entire BF^lh^ fascicles are too large for the field of view, and thus, an estimation of fascicle length is required through equation (Kellis et al., 2009).

Eccentric training appears to elicit greater increases in muscle CSA than concentric or traditional resistance training (Douglas et al., 2017). The mechanism seems to be a high level of mechanical tension per active motor unit (Prilutsky, 2000), stretch-induced strain (Toigo and Boutellier, 2006), and a greater propensity for exercise-induced muscle damage (McHugh, 2003), which may stimulate the hypertrophic signaling response to a greater extent. Increasing distal muscle hypertrophy with eccentric training results in increasing muscle CSA via the addition of sarcomeres in series in contrast to the addition of sarcomeres in parallel with concentric training (Franchi et al., 2014). This fact can explain the increase in fascicle angle after the eccentric (Duclay et al., 2008; Leong et al., 2014) as well as concentric training (Franchi et al., 2017). Simultaneously, stretch-induced strain from eccentric contractions, sensed within the Z-line region of titin, appears to elicit a specific anabolic signaling response (Franchi et al., 2017).

The influence of NHE on muscle architecture in our review was investigated in 5 of 11 articles (Lovell et al., 2018; Vianna et al., 2021; Medeiros et al., 2020; Mendiguchia et al., 2020; Siddle et al., 2024). Four included articles examined the effect of high volume in NHE, and one article (Siddle et al., 2024) examined the effect of the low volume protocol. Previous studies that investigated the effect of low volume on muscle architecture were mostly conducted on recreationally active males but not elite athletes (Presland et al., 2018). A similar exercise program (2–4 sets of 6–12 reps) was used across the studies where high volume has been presented. The difference was only in program duration (6–12 weeks). Only one study used a protocol with a low volume program (144 reps in 8 weeks) (Siddle et al., 2024).

Fascicle length of BF^lh^ is the most examined variable regarding the muscle architecture assessment. Recent studies examined that possessing BF^lh^ fascicles <10.56 cm significantly increased the future risk of HSI ∼4 times in elite Australian soccer players (Timmins et al., 2016). On the other hand, an increase in FL of 0.5 cm decreases the risk of hamstring strain by ∼ 74% on average. The influence of a high volume training program on this variable was present in 4 of 5 studies (Lovell et al., 2018; Vianna et al., 2021; Medeiros et al., 2020; Mendiguchia et al., 2020), while only one study used a low volume training program (Siddle et al., 2024). A significant increase by + 6.4% has been found after 8 weeks (456 reps) in elite female players (Vianna et al., 2021). An small increase of FL was found in professional male players with the same program and duration with low (1x per week) and high training frequency (2x per week) (Medeiros et al., 2020). This variable also increased by +7.38% after 6 weeks in the elite soccer players (Mendiguchia et al., 2020) and by +1.58% (small ES) after 12 weeks in amateur soccer players (Lovell et al., 2018) performing NHE before training sessions but not after. Low volume NHE programs elicit significant increases in FL of BF^lh^ after 6 weeks in recreational athletes with no previous NHE experience (Presland et al., 2018), while no meaningful difference between the low and high volume groups has been found. Contrary to that, no significant change was found after the low volume intervention in elite male academy soccer players (Siddle et al., 2024). One possible explanation is that the protocol has been done on elite young soccer players with previous exposure to NHE; therefore, to elicit significant improvements, the exercise intensity had to be higher (Siddle et al., 2024). Low volume also increased the FL of BF^lh^ and SM after 6 weeks in elite youth soccer players, but this change can be influenced by the inclusion of the second exercise (bilateral stiff-leg deadlift), which is more hip dominant movement and elicits greater activation of BF^lh^, opposite to NHE (knee dominant movement), where more ST muscle activity has been presented (Bourne et al., 2017).

Three of our included studies (Lovell et al., 2018; Mendiguchia et al., 2020; Medeiros et al., 2020) investigated the influence of high volume NHE on the MT. Small and medium increases in MT were presented after 6 and 8 weeks, respectively (Mendiguchia et al., 2020; Medeiros et al., 2020). The same result was presented with the group performing the NHE after, but not before, the soccer session (Lovell et al., 2018), where the compliance rate was presented below 50% in all groups; interestingly, this fact did not affect the training outcome as stated previously with higher volumes of NHE (Chesterton et al., 2021; Chebbi et al., 2022). This is not in line with results from the meta-analysis by Cuthbert et al. (2020), where the authors found no meaningful changes in MT. The authors assume that the problem can be in low training duration (8 weeks = <), though this did not affect the training outcome in the study where the lower training duration (6 weeks) has been implemented (Mendiguchia et al., 2020). On the contrary, MT of SM, ST, and BFlh did not change after the low volume program (Siddle et al., 2024), which is also probably influenced by lower intensity of exercise and application of programme on previously NHE trained players.

PA increased after a high volume intervention program in duration of 8 weeks in “after training group” (small ES) (Lovell et al., 2018) and 6 weeks (ES = 0.41) (Mendiguchia et al., 2020). No significant improvements were found in the angle of pennation after 8 weeks in high volume groups (Medeiros et al., 2020) and “before training group” (Lovell et al., 2018). Similarly, no significant difference in PA was found after low volume intervention with 8 weeks duration (Siddle et al., 2024). High volume resistance training is primarily responsible for increasing PA and CSA (Cuthbert et al., 2020). A decrease in PA as a desired outcome after eccentric resistance training (Gérard et al., 2020) was presented in meta-analysis by Cuthbert et al. (2020) in both low and high volume groups. On the other hand, from our review, we observed only a trivial increase in the angle of pennation after high volume training, while no changes were seen after low volume training application. The increase results in a higher physiological CSA with the addition of myofibrils in parallel. This essentially improved force transmission through the muscle-tendon unit and a higher architectural gear ratio (Douglas et al., 2017; Azizi and Roberts, 2014). The ratio allows the pennate biceps femoris to limit the strain from active fascicles, which results in protection during fast-velocity lengthening actions (Azizi and Roberts, 2014). Mechanical tension and intramuscular metabolic stress determine the hypertrophic signal of the muscle during high-intensity resistance training (Douglas et al., 2017). Although the mechanical tension applies in both types of volumes, low volume of NHE seem to not elicit the intramuscular metabolic stress enough, resulting in no hamstring architecture changes.

Despite the methodological differences in our review (total volumes, duration, playing levels), the high volume of NHE seems to increase the FL of hamstring muscles and MT with trivial but non significant changes observed in PA. Contrary to this, low-volume protocol yields no significant adaptations on these parameters. Intensity of exercise is a significant factor that must be considered when the goal is to further elicit the outcomes in previously trained players. We emphasize that more research must be done on this particular topic because of the lack of literature investigating the effect of low-volume NHE protocols on hamstring architecture in soccer players. Summary of the gaps, limitations and suggestions for the future studies are mentioned in Table 4.

**TABLE 4:** Research gaps identified in the literature and suggestions for future studies.

Gaps and limitations in the literature	Suggestions for future studies
Lack of studies comparing the effects of low and high volume on hamstring architecture in NHE experienced players	Investigate the effect of low volume on hamstring architecture for NHE experienced players
Lack of studies comparing the effects of low and high volume on eccentric strength NHE in experienced players	Compare the effects of low and high volumes of NHE on eccentric strength in experienced players
Variability of results in changes of peak eccentric torque after NHE when measuring on isokinetic device	Similarity of exercise as a measurement variable and training intervention in future studies (i.e., Nordic curl = NordBord; Knee flexion exercises = Isokinetic device) Standardization of assessment tools and outcome measures on isokinetic dynamometry (velocity, position) for future comparisons

## 5 Conclusion

This systematic review and meta-analysis critically examined the effects of low and high volume NHE protocols on eccentric strength and hamstring muscle architecture among soccer players. The eleven included studies revealed greater effectiveness in muscle architecture variables such as fascicle length and muscle thickness after high than low volume protocols, while no changes were seen between groups. Given the lack of evidence regarding the effect of low volume NHE on hamstring architectural adaptations in soccer players, further research is needed. Furthermore, there is no difference in eccentric peak force between high and low volume NHE, however the effect of both types of training on eccentric peak torque seems to be inconsistent in findings. The small to none effect of NHE on eccentric peak torque measured by the isokinetic device is likely influenced by testing conditions (e.g., angular velocity, body position) and non-similarity with the training exercise. The variability in assessment methods, particularly between NordBord and isokinetic dynamometry, introduces challenges in comparing eccentric strength outcomes. Factors such as different player levels (amateur, semi-professional, or professional), previous experience with NHE, and compliance with exercise significantly influence the training outcomes and must be taken into consideration. Therefore, standardization of assessment tools and outcome measures is critical for future comparisons on the effect of low and high volume of NHE on hamstring eccentric strength. Further research is also needed to determine the effect of low volume of NHE on the hamstring architecture adaptations regarding previous experience with NHE and player level.

### 5.1 Practical applications

We suggest that coaches may prioritize the high volume of NHE in the pre-season period in order to improve architectural adaptation of the hamstring muscles and, when starting with novice athletes, to increase compliance and decrease DOMS. Novice athletes should first use low-intensity exercise (i.e., band-assisted NHE, decreasing ROM) and gradually increase the intensity to perform exercise in full range of motion. Low volume of exercise can be used within the in-season period, where a high match schedule is presented to decrease compliance with exercise. Progressive overload must be used in experienced players with the aim to gradually increase the hamstring strength and architecture of hamstring muscles. Additionally, coaches should try to find the minimal effective dose of NHE, respecting the desired outcomes of exercise, compliance, and strength level of the players.

## Data Availability

The original contributions presented in the study are included in the article/supplementary material, further inquiries can be directed to the corresponding author.
